# Association Between Domestic Water Hardness and MRI-Based Measures of Brain Volume: Evidence from the UK Biobank

**DOI:** 10.34133/hds.0469

**Published:** 2026-10-06

**Authors:** Qi Sun, Li Yu, Ruixue Liu, Xianyu Zhu, Junda Qu, Zhixiang Wang, Liping Zhao, Han Lv

**Affiliations:** ^1^ Department of Radiology, Beijing Friendship Hospital, Capital Medical University, Beijing 100050, China.; ^2^ Precision and Intelligence Medical Imaging Lab, Beijing Friendship Hospital, Capital Medical University, Beijing 100050, China.

## Abstract

**Background:** While water hardness is a known factor in cardiovascular health, its influence on brain structure is not well understood. This study aimed to investigate the association between water hardness and brain volume. **Methods:** This prospective cohort study was performed with data from the UK Biobank. To assess the associations between water hardness and standardized volumes of total brain volume (TBV), white matter, and gray matter, generalized linear models were utilized. Potential nonlinear association between water hardness and brain volume were indicated by restricted cubic splines. **Results:** Compared with soft water (0 to 60 mg/l), hard water (>180 mg/l) correlated with reduced TBV (β: −0.06, 95% confidence interval [CI]: −0.09 to −0.04), white matter (β: −0.07, 95% CI: −0.09 to −0.04), and gray matter (β: −0.04, 95% CI: −0.06 to −0.02), with all *P* < 0.001. Restricted cubic spline analysis revealed linear relationships between water hardness and TBV. **Conclusions:** Elevated water hardness is associated with reduced brain volume. These findings suggest that optimizing drinking water mineral content might be a strategy for neuroprotection.

## Introduction

Water hardness, defined by its calcium (Ca) and magnesium (Mg) ion content, has been associated with various public health outcomes. Evidence suggests that these minerals play roles in cardiovascular health and glucose metabolism [1,2]. Population-based studies consistently report inverse correlations between chronic hard water consumption (>120 mg/l as CaCO₃) and the incidence of hypertension [3]. Mechanistically, this protection may stem from Ca-mediated vascular smooth muscle relaxation and Mg-dependent enhancement of insulin receptor signaling [4]. However, while systemic health effects have been investigated [5], the possible influence of long-term hard water intake on neurological health has not been extensively studied.

Brain volume is a robust neuroimaging biomarker reflecting neurological health [6]. This measure is also linked to cognitive functions such as memory and behavioral control [7]. Considering the worldwide increase in the aging population and the rise of neurodegenerative diseases [8], identifying environmental and lifestyle factors that influence brain health is critical. Although studies have examined diet [9], air pollution [10], and lifestyle factors [11,12], the role of mineral intake from drinking water has received limited attention.

Existing evidence on mineral intake and brain health is inconsistent. Some studies suggest that deficiencies in Ca and Mg could contribute to cognitive decline [13], but these have mainly focused on dietary supplements rather than drinking water. This distinction is important because waterborne minerals often have higher bioavailability than those from food [14]. Additionally, translational research indicates that such deficiencies may promote neuroinflammation, potentially leading to brain atrophy and neurodegeneration [15]. Despite this preliminary evidence, the association between drinking water-derived minerals and human brain structure has yet to be explored in large-scale population cohorts.

The UK Biobank boasts extensive neuroimaging data coupled with comprehensive records of environmental exposures [16], offering a unique opportunity to explore this relationship. This study aims to examine the link between water hardness and brain volume by leveraging this comprehensive dataset. It is hypothesized that elevated water hardness is associated with preserved total and regional brain volumes, potentially due to calcium/magnesium-dependent neuroprotective mechanisms. By integrating geospatial water quality data with multimodal magnetic resonance imaging (MRI) metrics, the findings may inform public health strategies to optimize drinking water standards for brain health.

## Methods

### Data sources and study design

This prospective cohort study leveraged data from the UK Biobank (Application ID: 105978). The UK Biobank constitutes a major prospective cohort resource that seeks to improve the prevention, diagnosis, and treatment of various diseases. This cohort recruited 502,412 individuals aged 40 to 69 years across the United Kingdom between 2006 and 2010. It collects extensive health-related data, including biological specimens, imaging data, environmental and lifestyle information, making it a valuable resource for investigating the links between environmental factors and health outcomes [17]. Ethical approval was granted by the Northwest Multi-Centre Research Ethics Committee. Written informed consent for data to be used in this research was obtained from all participants.

In this study, individuals were included if they had complete data on domestic water hardness, brain imaging metrics (MRI), and relevant covariates. A total of 456,200 participants were removed from the initial cohort for lacking either water hardness or MRI data, leaving 45,928 participants eligible for further screening. An additional 1,669 participants were excluded due to a diagnosis of neurological diseases, specifically Parkinson’s disease (*n* = 140), dementia (*n* = 131), stroke (*n* = 811), epilepsy (*n* = 410), and multiple sclerosis (*n* = 177), resulting in 44,259 participants. In the final step, 10,076 participants were excluded due to missing key covariates: Townsend deprivation index (*n* = 34), qualification (*n* = 595), income (*n* = 3,727), smoking status (*n* = 62), alcohol consumption (*n* = 7), physical activity (*n* = 5,567), body mass index (BMI, *n* = 37), cardiovascular disease (CVD, *n* = 20), and diabetes (*n* = 27). Consequently, 34,183 individuals were included in this study. Figure 1 illustrates the participant selection flowchart. A comparison of characteristics between participants included in and excluded from the analysis is presented in Table S1.

**Fig. 1.**
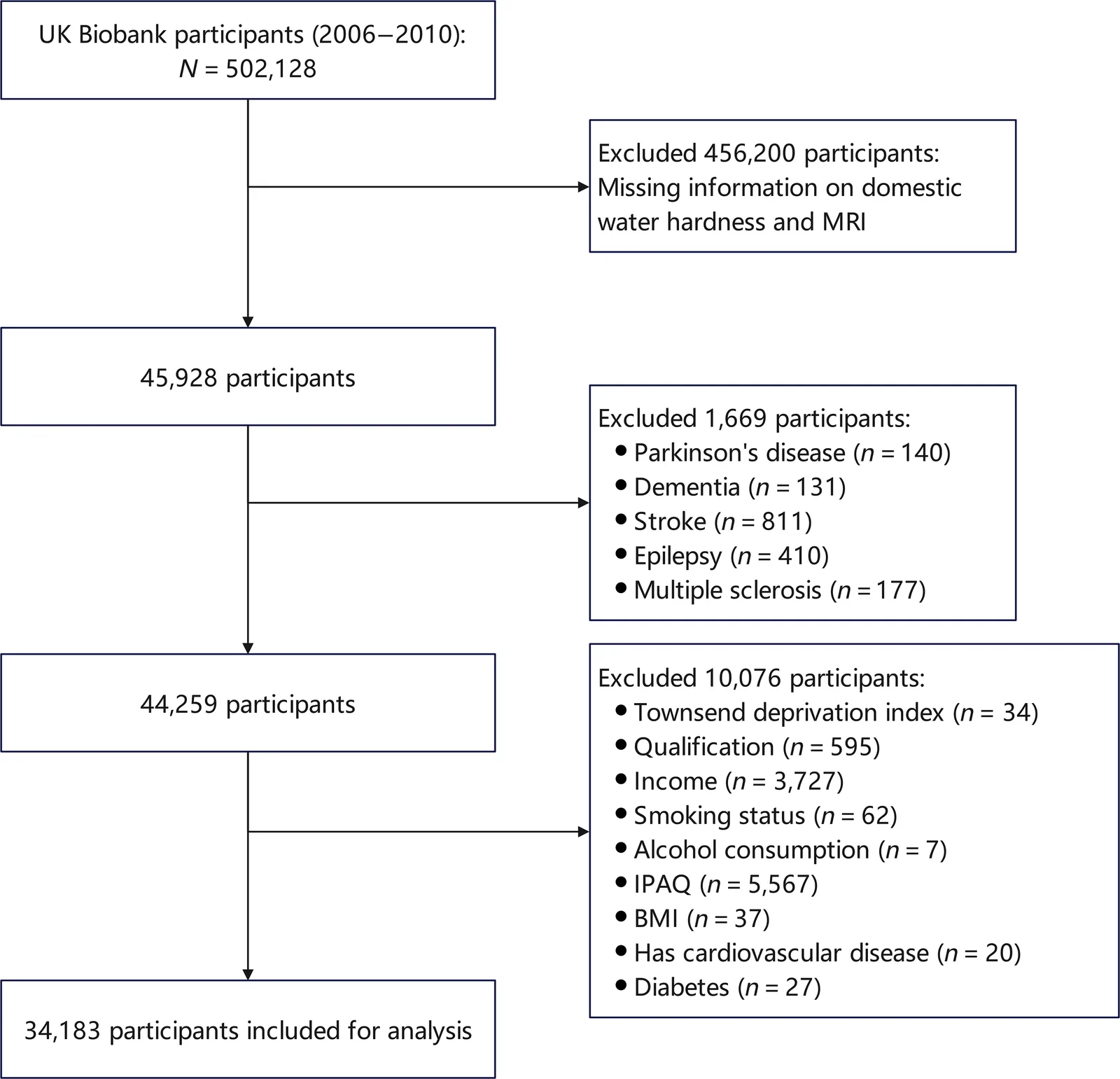
Flowchart of study. IPAQ, International Physical Activity Questionnaire; UK Biobank: United Kingdom Biobank; BMI, body mass index; MRI, magnetic resonance imaging.

### Water hardness exposure

In this analysis, water hardness data were obtained based on participants’ home addresses collected at the UK Biobank baseline assessment (2006 to 2010) [1]. These data were linked to local water supply information provided by utility companies across England, Wales, and Scotland. Specifically, the water hardness measures represent the average concentrations for the year 2005, which aligns with the start of the baseline recruitment. Although water hardness is primarily determined by local geology and is generally considered stable over time, using 2005 data for the entire follow-up period assumes temporal stability of water mineral content. Participants’ postcodes, which serve as shorthand for residential addresses, were used to match individual residences with their respective water supply zones. Typically, a postcode (comprising 5 to 7 alphanumeric characters) covers an average of 15 households, with a range of 1 to 100.

The water hardness data included concentrations of calcium carbonate (CaCO₃), calcium, and magnesium (all in mg/l), with CaCO₃ concentration used as the primary measure of water hardness. Water hardness was stratified via 2 separate methods. First, the US Geological Survey guidelines were followed, and 4 groups were established: soft (0 to 60 mg/l), moderately hard (>60 to 120 mg/l), hard (>120 to 180 mg/l), and very hard (>180 mg/l) [18]. Second, a binary split was applied based on the World Health Organization (WHO) limit for scale deposition, distinguishing between soft (<200 mg/l) and hard (≥200 mg/l) water [19].

### Outcome definition

Brain volume metrics included gray matter (GM) volume, white matter (WM) volume, total brain volume (TBV), and regional GM volumes, all measured in 2014 through MRI scans. A total of 122 regional GM volumes were analyzed, representing a subset of the 139 image-derived phenotypes provided by the UK Biobank [20]. These phenotypes were generated using the standard UK Biobank image processing pipeline. Briefly, FMRIB’s Automated Segmentation Tool was used to segment T1-weighted images into distinct tissue types [21]. Regional GM volumes were calculated by summing the GM partial volume estimates within regions of interest defined in MNI152 space. Their definitions were generated by combining the Harvard–Oxford atlas (for both cortical and subcortical regions) [22] and the Diedrichsen cerebellar atlas [23]. The selected 122 regions cover the entire cerebral cortex, subcortical structures, and major cerebellar lobules, while 17 minor cerebellar subregions were not included in the current dataset. The imaging data were processed following a standardized analytical pipeline to ensure consistency and reliability [24].

### Covariate definitions

The study incorporated the following covariates: age (years), measured at recruitment; sex (male, female); ethnicity (White, Other). Assessment of socioeconomic status was evaluated using the Townsend deprivation index, and participants were classified into low, medium, or high groups according to population quintiles [25]. Stratification of educational qualification was based on the highest level attained (nonuniversity, university degree). Employment status was classified into 3 groups: employed, retired, or other. Average household income was categorized into 3 groups based on reported ranges: less than £30,999, £31,000 to £100,000, and greater than £100,000. For smoking, participants were determined as never, former, or current smokers. Alcohol consumption frequency was grouped into the categories of frequent (3 to 4 times per week), occasional (once or twice per week), or never. Physical activity was evaluated via the International Physical Activity Questionnaire and classified according to metabolic equivalent of task (MET) minutes per week, resulting in 3 levels: low (<600 MET min/wk), moderate (600 to 3,000 MET min/wk), and high (>3,000 MET min/wk) [26]. Standard BMI categories were applied: normal weight (<25 kg/m^2^), overweight (≥25 kg/m^2^), and obese (>29.9 kg/m^2^). The assessment of CVD and diabetes was defined by self-reported diagnoses provided by a doctor (yes, no). In this context, CVD was defined by the occurrence of heart attack, angina, stroke, or hypertension. Computation of a healthy diet score involved 7 food groups: fish, processed meat, unprocessed red meat, fruits, vegetables, refined grains, and whole grains. Each component is scored based on specific intake criteria: fish (≥2 servings/week), processed meat (≤1 serving/week), red meat (≤1.5 servings/week), fruits (≥3 servings/d), vegetables (≥3 servings/d), refined grains (≤1.5 servings/d of white bread), and whole grains (≥3 servings/d of whole grains). A point was awarded for each criterion met, resulting in a total score ranging from 0 to 7. A score of ≥4 indicated a healthy diet, while a score of <4 indicated an unhealthy diet [27]. Covariates, including BMI, alcohol consumption, physical activity, and diet, were derived from data collected at the baseline assessment. Baseline measures were selected to ensure sufficient sample size and to represent long-term physiological and lifestyle profiles preceding the neuroimaging outcome.

### Statistical analysis

Population characteristics were summarized via descriptive statistics. Means ± SD were used to present continuous variables, while frequencies and percentages were utilized for categorical variables.

To facilitate comparison of effect sizes, all continuous variables (including water hardness concentrations, brain volumes, and continuous covariates) were standardized using *z*-scores prior to the primary analysis. To investigate the relationships between water hardness and brain volume metrics, generalized linear models (GLMs) were employed, adjusting for potential confounders including demographic, socioeconomic, and health-related factors. Results were expressed as standardized regression coefficients (β) and 95% confidence intervals (CIs). Control for the false discovery rate (FDR) was achieved using the Benjamini-Hochberg procedure to account for multiple testing across the 122 regional GM volumes. The FDR correction was applied separately for each water hardness measure (exposure) across all neuroimaging outcomes.

Subgroup analyses were performed to investigate potential effect modification by age, sex, ethnicity, and lifestyle factors. Specific associations between water hardness and brain volumes were estimated by constructing separate GLMs for each subgroup. To assess the statistical significance of subgroup differences (interaction effects), models with and without the multiplicative interaction term (water hardness × subgroup variable) were compared using likelihood ratio tests.

Restricted cubic spline (RCS) models explored nonlinear associations between water hardness and brain volume. To balance flexibility and overfitting, knot selection was driven by the distribution of each exposure variable. Specifically, for CaCO₃ and Ca, 4 knots were positioned at the 5th, 35th, 65th, and 95th percentiles. For Mg, given its discrete nature and limited range, 3 knots were assigned at the 10th, 50th, and 90th percentiles.

To verify the robustness of findings, several sensitivity analyses were conducted: (a) modeling water hardness as a continuous variable; (b) using WHO classifications of water hardness; (c) adjusting for the time interval between water hardness assessment and MRI acquisition; and (d) to mitigate potential bias from residential mobility, the sample was restricted to participants with stable residence (defined as duration of residence at current address ≥ time since baseline) to address potential bias from residential mobility.

R software (version 4.3.2) was conducted for all statistical analysis, and a *P* value < 0.05 was considered statistically significant.

## Results

### Participant characteristics

A total of 34,183 participants were included in this analysis (Table 1), categorized into 4 groups based on water hardness levels: 0 to 60 mg/l (*n* = 15,677), 60 to 120 mg/l (*n* = 7,992), 120 to 180 mg/l (*n* = 1,504), and >180 mg/l (*n* = 9,010). Age varied across groups, with participants exposed to >180 mg/l water being slightly older (median, 56.00 years; interquartile range: 49.00 to 61.00) compared to those exposed to 0 to 60 mg/l (median, 55.00 years; interquartile range: 48.00 to 60.00; *P* < 0.001). Socioeconomic status, education level, and alcohol consumption also showed notable differences (*P* < 0.001 for all). Conversely, no significant variation was detected in sex distribution across different water hardness categories (*P* = 0.849).

**Table 1.** Baseline characteristics of participants

		Water hardness				*P*
	Level	0–60 mg/l	60–120 mg/l	120–180 mg/l	>180 mg/l	
*n*		15,677	7,992	1,504	9,010	
Age (year), median [IQR]		55.00 [48.00, 60.00]	55.00 [49.00, 60.00]	55.00 [48.00, 60.00]	56.00 [49.00, 61.00]	<0.001
Sex (%)	Male	7,823 (49.9)	3,981 (49.8)	748 (49.7)	4,444 (49.3)	0.849
	Female	7,854 (50.1)	4,011 (50.2)	756 (50.3)	4,566 (50.7)	
Ethnicity (%)	Others	306 (2.0)	160 (2.0)	44 (2.9)	513 (5.7)	<0.001
	White	15,371 (98.0)	7,832 (98.0)	1,460 (97.1)	8,497 (94.3)	
Socioeconomic status (%)	Low	2,825 (18.0)	1,291 (16.2)	235 (15.6)	2,429 (27.0)	<0.001
	Medium	9,521 (60.7)	5,105 (63.9)	902 (60.0)	4,988 (55.4)	
	High	3,331 (21.2)	1,596 (20.0)	367 (24.4)	1,593 (17.7)	
Qualification category (%)	Nonuniversity	7,798 (49.7)	4,565 (57.1)	724 (48.1)	4,099 (45.5)	<0.001
	University	7,879 (50.3)	3,427 (42.9)	780 (51.9)	4,911 (54.5)	
Employment status (%)	Employed	11,226 (71.6)	5,692 (71.2)	1,082 (71.9)	6,313 (70.1)	0.008
	Others	782 (5.0)	404 (5.1)	87 (5.8)	543 (6.0)	
	Retired	3,669 (23.4)	1,896 (23.7)	335 (22.3)	2,154 (23.9)	
Income (%)	High	1,150 (7.3)	521 (6.5)	111 (7.4)	1,098 (12.2)	<0.001
	Low	5,163 (32.9)	2,761 (34.5)	469 (31.2)	2,543 (28.2)	
	Medium	9,364 (59.7)	4,710 (58.9)	924 (61.4)	5,369 (59.6)	
Smoking status (%)	Current	989 (6.3)	464 (5.8)	97 (6.4)	575 (6.4)	0.139
	Never	9,539 (60.8)	4,912 (61.5)	916 (60.9)	5,358 (59.5)	
	Previous	5,149 (32.8)	2,616 (32.7)	491 (32.6)	3,077 (34.2)	
Alcohol consumption (%)	Frequent	8,124 (51.8)	4,017 (50.3)	796 (52.9)	4,892 (54.3)	<0.001
	Never	656 (4.2)	318 (4.0)	69 (4.6)	398 (4.4)	
	Occasional	6,897 (44.0)	3,657 (45.8)	639 (42.5)	3,720 (41.3)	
IPAQ (%)	High	6,210 (39.6)	3,192 (39.9)	585 (38.9)	3,544 (39.3)	0.040
	Low	2,875 (18.3)	1,468 (18.4)	311 (20.7)	1,568 (17.4)	
	Medium	6,592 (42.0)	3,332 (41.7)	608 (40.4)	3,898 (43.3)	
BMI (%)	Normal	6,055 (38.6)	2,818 (35.3)	609 (40.5)	3,673 (40.8)	<0.001
	Overweight	6,836 (43.6)	3,546 (44.4)	619 (41.2)	3,804 (42.2)	
	Obese	2,786 (17.8)	1,628 (20.4)	276 (18.4)	1,533 (17.0)	
Cardiovascular disease (%)	No	12,515 (79.8)	6,298 (78.8)	1,191 (79.2)	7,208 (80.0)	0.197
	Yes	3,162 (20.2)	1,694 (21.2)	313 (20.8)	1,802 (20.0)	
Diabetes (%)	No	15,311 (97.7)	7,776 (97.3)	1,468 (97.6)	8,796 (97.6)	0.358
	Yes	366 (2.3)	216 (2.7)	36 (2.4)	214 (2.4)	
Healthy diet (%)	No	7,013 (44.7)	3,639 (45.5)	648 (43.1)	3,835 (42.6)	<0.001
	Yes	8,664 (55.3)	4,353 (54.5)	856 (56.9)	5,175 (57.4)	

IQR, interquartile range; *n*, number of participants; BMI, body mass index; IPAQ, International Physical Activity Questionnaire; CVD, cardiovascular disease; *P*, *P* value. The Kruskal–Wallis test and the chi-square test were applied to analyze continuous and categorical variables, respectively.

### Associations between water hardness and brain volumes

Table 2 indicates the link between water hardness and brain volumes (TBV, WM, and GM). Compared to the reference group (0 to 60 mg/l), higher water hardness levels showed significant associations with decreased brain volumes. Specifically, participants residing in areas with water hardness >180 mg/l had significantly lower TBV volume (β: −0.06; 95% CI: −0.09, −0.04), WM (β: −0.07; 95% CI: −0.09, −0.04), and GM (β: −0.04; 95% CI: −0.06, −0.02), with all associations achieving statistical significance at *P* < 0.001. Among participants in moderately hard water areas (60 to 120 mg/l), a significant reduction was found in TBV volume (β: −0.04; 95% CI: −0.06, −0.02; *P* < 0.001), WM (β: −0.04; 95% CI: −0.06, −0.01; *P* = 0.004), and GM (β: −0.03; 95% CI: −0.05, −0.01; *P* = 0.012). The associations between water hardness and subcortical brain volumes are detailed in Table S2.

**Table 2.** Associations between water hardness and brain volumes based on generalized linear models

Variable		Volume of TBV			Volume of WM			Volume of GM		
		β (95% CI)	*P*	P_FDR	β (95% CI)	*P*	P_FDR	β (95% CI)	*P*	P_FDR
Water hardness	0–60 mg/l	Ref	Ref	Ref	Ref	Ref	Ref	Ref	Ref	Ref
	>60–120 mg/l	−0.04 (−0.06, −0.02)	<0.001	0.010	−0.04 (−0.06, −0.01)	0.004	0.037	−0.03 (−0.05, −0.01)	0.012	0.092
	>120–180 mg/l	0.03 (−0.02, 0.07)	0.212	0.481	0.01 (−0.04, 0.06)	0.622	0.807	0.03 (−0.01, 0.07)	0.119	0.366
	>180 mg/l	−0.06 (−0.09, −0.04)	<0.001	<0.001	−0.07 (−0.09, −0.04)	<0.001	<0.001	−0.04 (−0.06, −0.02)	<0.001	0.002
CaCO_3_		−0.02 (−0.03, −0.01)	<0.001	<0.001	−0.02 (−0.03, −0.01)	<0.001	<0.001	−0.01 (−0.02, −0.00)	0.023	0.070
Ca		−0.05 (−0.06, −0.05)	<0.001	<0.001	−0.07 (−0.08, −0.06)	<0.001	<0.001	−0.03 (−0.04, −0.02)	<0.001	<0.001
Mg		0.01 (−0.00, 0.02)	0.060	0.185	−0.00 (−0.01, 0.01)	0.457	0.631	0.02 (0.01, 0.02)	<0.001	0.003

β, regression coefficient; CI, confidence interval; *P*, unadjusted *P* value; P_FDR, false discovery rate adjusted *P* value; TBV, total brain volume (normalized); WM, white matter (normalized); GM, gray matter (normalized); CaCO_3_, calcium carbonate; Ca, calcium; Mg, magnesium. The reference group for water hardness is 0 to 60 mg/l. Separate statistical models were constructed for each exposure variable (water hardness categories, CaCO_3_, Ca, and Mg). All statistical models included adjustments for a comprehensive set of covariates: age, sex, ethnicity, socioeconomic status, educational attainment, employment status, income, smoking status, alcohol consumption, physical activity, BMI, cardiovascular disease, diabetes, and dietary patterns. Additionally, all reported brain volumes were scaled to account for head size.

Higher Ca levels were associated with significant decreases in TBV (β: −0.05; 95% CI: −0.06, −0.05), WM (β: −0.07; 95% CI: −0.08, −0.06), and GM (β: −0.03; 95% CI: −0.04, −0.02), all *P* < 0.001. Mg concentrations were positively associated with GM (β: 0.02; 95% CI: 0.01, 0.02; *P* < 0.001) but showed no significant associations with TBV or WM. Several subcortical structures associated with water hardness were identified in Fig. 2. Detailed findings are presented in Table S3.

**Fig. 2:**
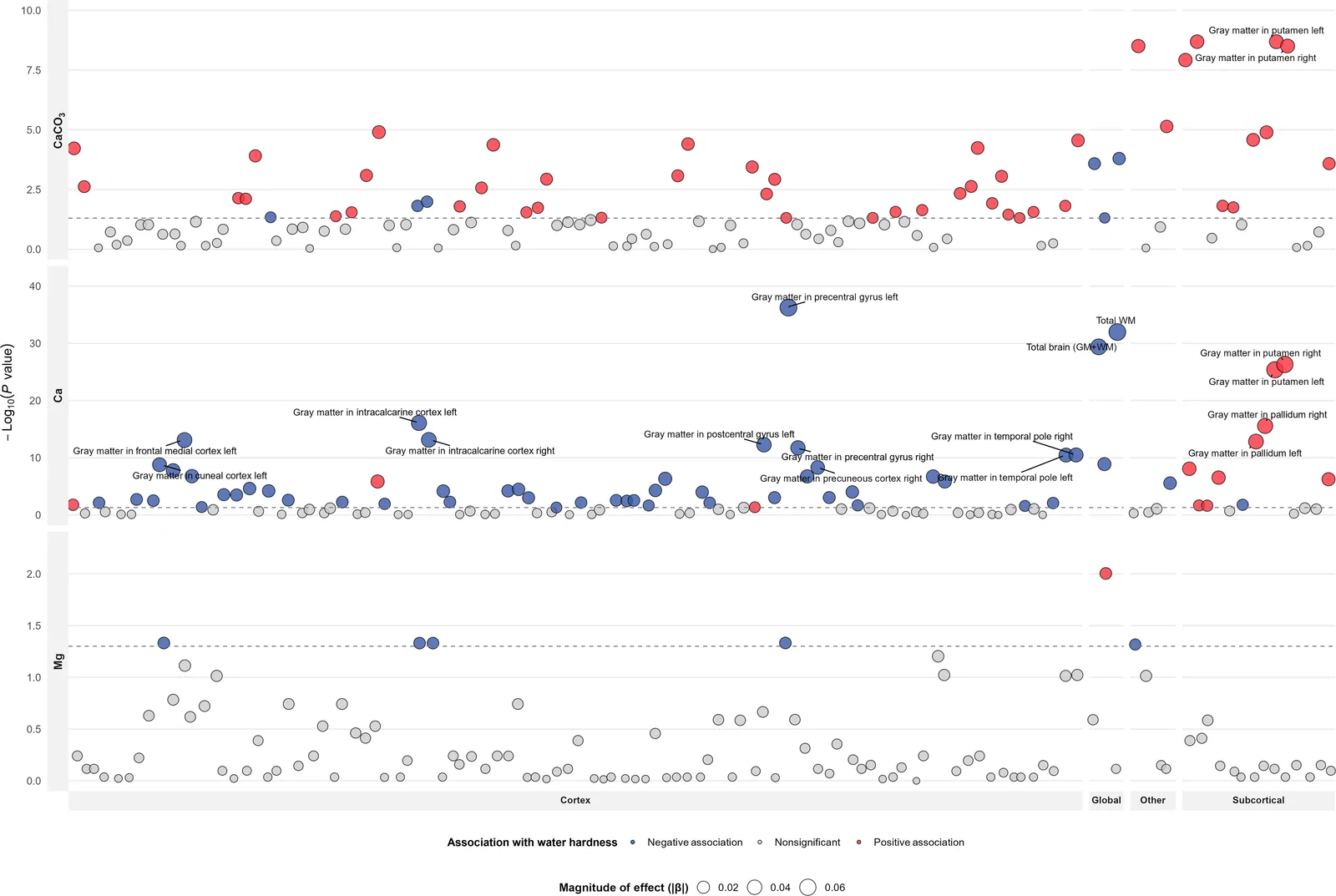
Associations between water mineral content (CaCO^3^, Ca, and Mg) and brain volume across different brain regions. The figure presents the associations between water mineral content (CaCO^3^, Ca, and Mg) and brain volumes across various brain regions. The y-axis represents the −log10(*P* value) indicating the strength of the association, while the x-axis represents brain volume. Dots above the dashed line represent significant associations (*P* < 0.05). The size of the dots reflects the magnitude of the effect, with larger dots indicating stronger associations.

The primary findings were further corroborated by multiple sensitivity analyses (Tables S3 to S6). First, modeling water hardness as a continuous variable yielded consistent results (Table S3). Second, applying WHO classifications for water hardness showed similar patterns (Table S4). Third, adjusting for the time interval between water hardness assessment and MRI acquisition did not materially change the associations (Table S5). Fourth, restricting the analysis to participants with stable residence demonstrated that the results were not driven by residential mobility (Table S6).

### Subgroup analysis

Subgroup analyses revealed differences in the association between water hardness and brain volume across various demographic and health-related factors (Table 3). A stronger negative association with higher water hardness (>180 mg/l) was detected in older participants (age >60 years: β = −0.12, 95% CI: −0.16 to −0.08), and a significant interaction between age and water hardness was confirmed (*P* < 0.001). In both males and females, high water hardness was linked to reduced brain volume (*P* interaction = 0.779). No statistically significant interactions were identified for ethnicity, smoking status, or BMI. Subgroup analyses of water hardness in relation to WM and GM are presented in Tables S7 and S8.

**Table 3.** Subgroup analysis of the association between water hardness and brain volume

Group		Water hardness			*P* interaction
		60–120 mg/l	120–180 mg/l	>180 mg/l	
Sex	Female	−0.04 (−0.07, −0.01)	0.05 (−0.01, 0.11)	−0.06 (−0.09, −0.03)	0.676
	Male	−0.03 (−0.07, −0.00)	0.01 (−0.05, 0.07)	−0.07 (−0.10, −0.04)	
Age	>60	−0.05 (−0.10, −0.01)	−0.01 (−0.10, 0.08)	−0.12 (−0.16, −0.08)	<0.001
	50–60	−0.05 (−0.09, −0.00)	0.03 (−0.05, 0.11)	−0.07 (−0.11, −0.03)	
	<50	−0.02 (−0.05, 0.01)	0.06 (−0.01, 0.13)	−0.06 (−0.09, −0.03)	
Ethnicity	White	−0.04 (−0.06, −0.01)	0.03 (−0.02, 0.07)	−0.06 (−0.09, −0.04)	0.900
	Other	−0.10 (−0.26, 0.05)	0.02 (−0.25, 0.28)	−0.08 (−0.20, 0.04)	
Smoking status	Never	−0.04 (−0.07, −0.01)	0.06 (0.01, 0.12)	−0.06 (−0.08, −0.03)	0.213
	Previous	−0.03 (−0.07, 0.01)	−0.05 (−0.12, 0.03)	−0.07 (−0.10, −0.03)	
	Current	−0.02 (−0.11, 0.08)	0.04 (−0.14, 0.21)	−0.10 (−0.18, −0.01)	
Alcohol consumption	Occasional	−0.05 (−0.08, −0.02)	0.03 (−0.03, 0.09)	−0.09 (−0.12, −0.06)	0.241
	Frequent	−0.02 (−0.06, 0.01)	0.01 (−0.05, 0.08)	−0.03 (−0.07, −0.00)	
	Never	−0.05 (−0.16, 0.07)	0.10 (−0.11, 0.31)	−0.03 (−0.14, 0.07)	
BMI	Normal	−0.03 (−0.06, 0.01)	0.03 (−0.03, 0.10)	−0.04 (−0.08, −0.01)	0.283
	Overweight	−0.04 (−0.07, −0.00)	0.01 (−0.06, 0.08)	−0.09 (−0.13, −0.06)	
	Obese	−0.05 (−0.10, −0.01)	0.05 (−0.05, 0.15)	−0.05 (−0.10, 0.00)	
Has cardiovascular disease	No	−0.03 (−0.05, −0.00)	0.04 (−0.00, 0.09)	−0.06 (−0.09, −0.04)	0.242
	Yes	−0.07 (−0.12, −0.02)	−0.04 (−0.14, 0.05)	−0.06 (−0.11, −0.01)	
Diabetes diagnosed by doctor	No	−0.04 (−0.06, −0.01)	0.02 (−0.02, 0.07)	−0.06 (−0.08, −0.04)	0.616
	Yes	−0.10 (−0.24, 0.04)	0.15 (−0.13, 0.44)	−0.11 (−0.26, 0.04)	
Has healthy diet	No	−0.02 (−0.05, 0.01)	0.02 (−0.04, 0.07)	−0.07 (−0.10, −0.05)	0.168
	Yes	−0.05 (−0.09, −0.02)	0.04 (−0.02, 0.11)	−0.05 (−0.08, −0.02)	

β, regression coefficient; CI, confidence interval; BMI, body mass index; *P* interaction, *P* value for interaction. Subgroup analyses were conducted across categories of sex, age, ethnicity, smoking status, and alcohol consumption, BMI, cardiovascular disease, diabetes status, and diet quality. Interaction *P* values were derived by performing​ the likelihood ratio rest (LRT), conducted by​ a comparison between nested models that included versus excluded the interaction term.

### RCS analysis

The RCS models showed significant associations between water mineral content and brain volumes (Fig. 3). For CaCO₃, the relationship with TBV was linear, indicating a continuous decline as water hardness increased. In contrast, a nonlinear trend was observed for Ca (*P* < 0.05 for TBV and GM), where brain volumes remained relatively stable up to 50 mg/l, followed by a marked decline. For Mg, a distinct nonlinear inverted U-shaped association was identified (*P* < 0.001), with brain volumes peaking at approximately 4.5 mg/l before decreasing at higher concentrations.

**Fig. 3:**
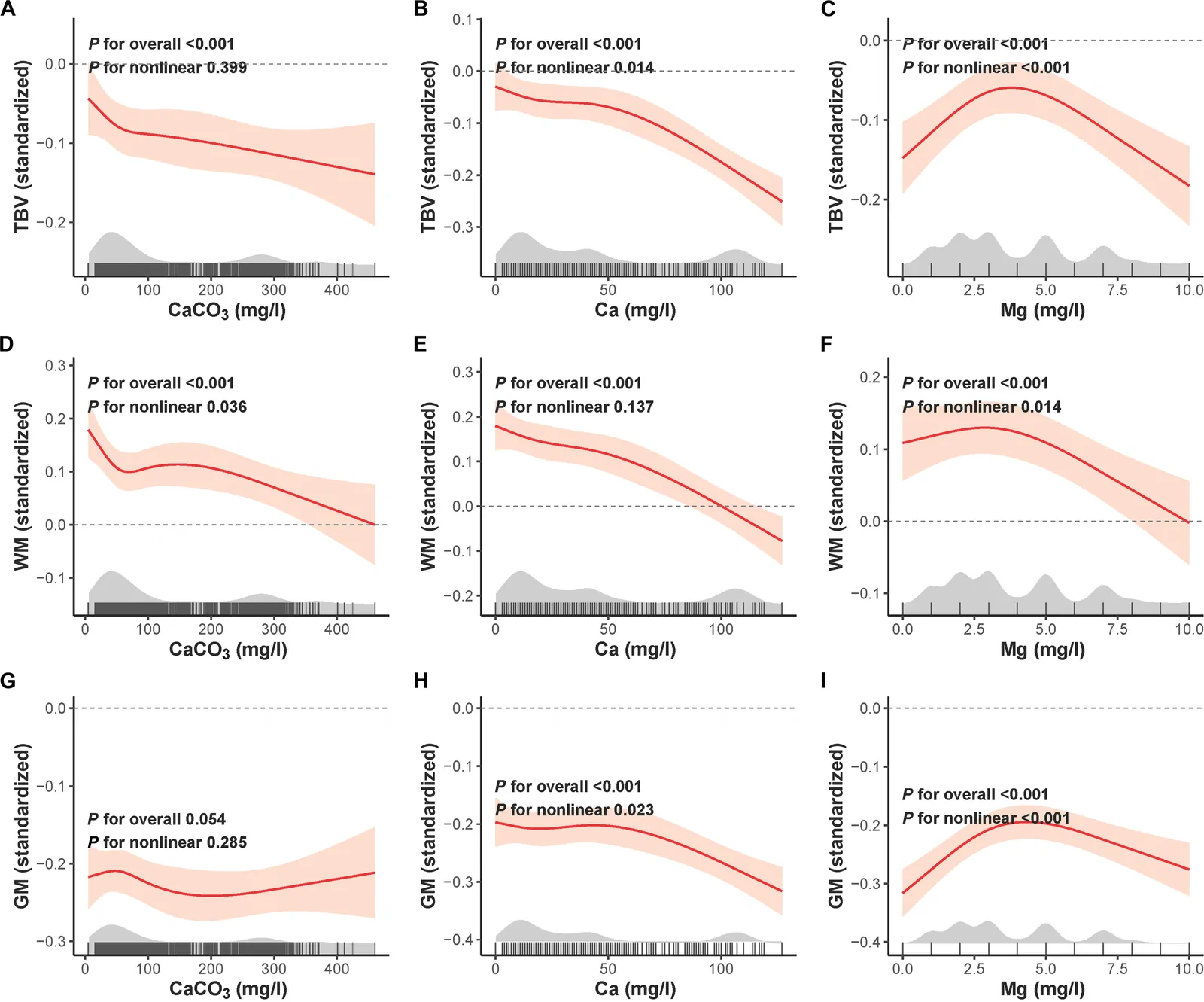
Restricted cubic spline analysis of the association between drinking water hardness components and brain volumes. Dose–response relationships between water hardness components (columns: CaCO_3_, calcium, and magnesium) and standardized brain volumes (rows: total brain volume [A to C], white matter [D to F], gray matter [G to I]). The solid red lines represent the multivariable-adjusted regression estimates, and the shaded red areas indicate the 95% confidence intervals. The gray shaded areas at the bottom display the density distribution of the water hardness concentrations. All models were adjusted for age, sex, BMI, ethnicity, socioeconomic status, qualification, employment, income, smoking, alcohol consumption, cardiovascular disease, physical activity, diabetes, and healthy diet score. The dashed horizontal line indicates the reference value (*Y* = 0). *P* values for overall association and nonlinearity are shown in each panel. Ca, calcium; CaCO^3^, calcium carbonate; GM, gray matter volume; Mg, magnesium; WM, white matter volume.

## Discussion

Utilizing data from the UK Biobank, this study explored the link between water hardness and brain structural volumes by employing both GLM and RCS to assess linear and nonlinear relationships. The findings revealed that elevated concentrations of CaCO₃ and Ca were significantly correlated significantly with reductions in brain volumes, particularly TBV and GM, while Mg exhibited a positive association with brain volume.

This study provides the first systematic evidence linking water hardness to brain morphology. The GLM results showed that significant associations were found between water hardness and brain volumes, with higher levels of CaCO₃ and Ca being associated with reductions in TBV, GM, and WM. Previous research has largely focused on the cardiovascular effects of water hardness [2,28,29], with limited exploration of its impact on brain health. While studies have linked excessive Ca intake with vascular calcification [30], the specific influence of water hardness on brain structure has remained underexplored. Our results suggest that Ca in drinking water could contribute to brain atrophy, particularly in regions such as TBV and GM, which are critical for cognitive functioning. Findings of this study are consistent with prior study indicating that the risk of Alzheimer’s disease in the elderly may be increased by higher serum Ca levels [31]. The positive association observed with Mg aligns with prior research. The relationship between Mg and brain structure was assessed in a couple of recent research using neuroimaging [32,33]. In one UK Biobank cohort, increases in GM and hippocampal volumes were associated with dietary Mg intake [33]. In another, greater TBV and lobar volumes, along with reduced odds of subcortical infarcts, were linked to higher serum Mg [32]. These observations highlight the necessity of considering environmental factors like water quality in public health strategies aimed at preserving brain health and preventing neurodegenerative conditions.

Our findings presented an intriguing divergence from our initial hypothesis: While higher Mg levels were associated with larger GM, consistent with a neuroprotective role, higher Ca and total water hardness were linked to reduced brain volumes. This apparent contradiction may be accounted for by the contrasting biological effects of these minerals. Although Ca is essential for neuronal function, excessive intake or dysregulation, particularly in older adults, can contribute to vascular calcification and endothelial dysfunction, which are established risk factors for cerebral atrophy and small vessel disease [34–36]. In contrast, Mg functions as a natural Ca antagonist and plays a pivotal role in maintaining vascular tone, reducing oxidative stress, and preventing excitotoxicity [15,37]. Thus, the overall negative association observed with total water hardness (CaCO₃) likely reflects the dominance of Ca’s adverse vascular effects over Mg’s protective benefits in this population. Specifically, exposure to high water hardness (>180 mg/l) was associated with a 0.06 SD reduction in TBV, corresponding to an approximately 0.6% decrease. This magnitude of atrophy is comparable to approximately 1 to 2 years of normal brain aging (estimated at 0.2% to 0.5% annual loss) [6,38], highlighting the potential clinical relevance of long-term exposure to hard water.

Variation in the associations between water hardness and brain metrics across diverse demographic and health characteristics was revealed by subgroup analysis. Age was a significant modifier, with older participants (>60 years) showing the most pronounced reductions in brain volume at water hardness > 180 mg/l. The increased susceptibility of older adults to environmental risk factors affecting cognitive function has been documented in previous studies, lending support to this finding [39]. The absence of significant interaction by sex suggests that the relationship between water hardness and brain volume does not differ between men and women. Although previous studies have highlighted differences in cognitive decline between sexes [40], our findings indicate that the effect of water hardness on brain volume is consistent across genders. This suggests that water hardness exerts a similar influence on brain structure in both men and women, irrespective of the established cognitive differences between the sexes in other contexts. Similarly, subgroup analyses by ethnicity, smoking status, alcohol consumption, CVD, and diabetes revealed no significant interactions, indicating that the effect of water hardness on brain volume is broadly consistent across these factors. However, individuals with normal BMI showed stronger associations with brain volume reduction at high water hardness levels, suggesting that metabolic health might play a role in modulating these effects, despite the lack of statistical significance in the interaction term.

The RCS analysis refined the understanding of the dose–response relationships. For total water hardness, a continuous, linear decline in TBV was observed, suggesting that higher water hardness is generally detrimental. However, for Ca, a potential threshold effect was identified, with brain volumes remaining stable up to approximately 50 mg/l before declining. In contrast, Mg exhibited a distinct nonlinear profile characterized by an inverted U-shape, where neuroprotective effects were evident up to a peak of approximately 4.5 mg/l. Beyond this turning point, the association reversed. Prior studies have also identified nonlinear relationships between Mg and mild cognitive impairment, further supporting its complex role in brain health [41]. Other research has highlighted the intricate connection between dietary Mg intake and metabolic diseases [42]. These findings underscore the complexity of water mineral content’s effects on brain health, with differing impacts based on exposure levels.

While the observational methodology limits inferences regarding causal biological mechanisms, several potential pathways might underlie the observed associations. One hypothesis concerns vascular calcification. Cerebral vascular calcification may be promoted by elevated calcium concentrations in drinking water, which could subsequently compromise cerebral perfusion [34]. Such processes could theoretically lead to brain atrophy in vulnerable regions [43], a notion supported by studies linking arterial calcium deposition to cognitive decline [35]. Another potential pathway might involve oxidative stress, where dysregulation of Ca homeostasis could trigger cellular damage [44]. Conversely, the observed positive relationship of Mg with brain structural volumes might be attributed to its neuroprotective properties [45,46]. A pivotal role in the maintenance of neuronal integrity and the modulation of oxidative stress is played by Mg [37], suggesting that higher Mg levels could potentially support brain structure preservation. These findings point to a multifactorial influence of water hardness on brain health, and further investigation to clarify the underlying biological pathways. It should be noted that our study is observational, and the exact biological mechanisms need to be clarified. While we hypothesize that processes such as vascular calcification or Ca-induced neurotoxicity might contribute to the observed brain atrophy, these interpretations are tentative.

The present investigation is characterized by several distinctive merits. First, it profits from a large sample size and a well-characterized UK Biobank cohort, providing high-quality data on environmental exposures and brain imaging metrics. Additionally, the longitudinal design allowed for the evaluation of long-term water hardness exposure and its impact on brain volume over time. Furthermore, we used rigorous methods to adjust for numerous potential confounders, including lifestyle, socioeconomic status, and metabolic factors, ensuring more robust and reliable findings.

However, several limitations should be considered. First, water hardness was assigned based on utility data from 2005, while MRI scans were conducted later point (2014 onwards). This temporal lag introduces the potential for exposure misclassification, particularly for participants who moved residence during the follow-up period. Although water hardness is largely geologically determined and assumed to be time-stable, residential mobility could alter individual exposure. To address this, we performed a sensitivity analysis restricted to participants with stable residence, which yielded consistent findings, suggesting that mobility-related bias is minimal. Second, even after adjusting for various potential confounders, persistent confounding from unmeasured factors such as long-term dietary habits or other environmental exposures cannot be ruled out. Specifically, data on other water constituents (e.g., fluoride, chloride, and sulfate) or industrial contaminants, which could potentially correlate with water hardness and influence brain health, were lacking. Third, associations were attenuated when adjusting for assessment center, likely due to overadjustment given the strong collinearity between water hardness and geographic location. Therefore, adjustment for specific socioeconomic confounders was prioritized over broad regional indicators, although residual spatial confounding cannot be ruled out. Fourth, a comparison of baseline characteristics indicated potential selection bias, with included participants being generally younger, wealthier, and healthier than those excluded. This “healthy volunteer” bias, typical of the UK Biobank imaging subsample [47], may limit external generalizability to frailer populations but is unlikely to compromise internal validity given the comprehensive adjustment for covariates. Lastly, our reliance on postcode-based water hardness data fails to consider individual variations in drinking water behaviors or in-home water treatment methods. Furthermore, assigning water hardness based on postcodes may introduce measurement error, as postcodes can cover multiple addresses or supply zones. However, such misclassification is likely nondifferential with respect to brain volume, resulting in a potential dilution of the effect estimates.

## Conclusion

In summary, this study found that elevated drinking water Ca concentrations and hardness were associated with reduced brain volumes, while Mg showed a positive association. Further studies are required to replicate these findings and explore the specific biological pathways involved.

## Ethical Approval

This research was conducted in accordance with the ethical principles of the Declaration of Helsinki, approved by the Northwest Ethics Committee (REC 21/NW/0157). All participants provided informed consent.

## Data Availability

The data reported in this paper are available via application directly to the UK Biobank.
